# Examining presenteeism and productivity losses among nursing workers: a cross-sectional study

**DOI:** 10.21203/rs.3.rs-4739078/v1

**Published:** 2024-08-26

**Authors:** David Márcio de Oliveira Barreto, Normeíza Márcia Fonseca Barreto, Sanay Victorino de Souza, Andréa Costa de Andrade, Handerson Silva-Santos, Neha Reddy, Tatiane Araújo-dos-Santos, Ednir Assis Souza, Johis Ortega, Tatiane Cunha Florentino

**Affiliations:** School of Nursing, Universidade Federal Do Amazonas; School of Nursing, Universidade Federal Do Amazonas; School of Nursing, Universidade Federal Do Amazonas; School of Nursing, Universidade Federal Do Amazonas; School of Nursing, Universidade Federal da Bahia; School of Nursing and Health Studies, University of Miami; School of Nursing, Universidade Federal da Bahia; School of Nursing, Universidade Federal da Bahia; School of Nursing and Health Studies, University of Miami; School of Nursing, Universidade Federal da Bahia

**Keywords:** Presenteeism, Absenteeism, Nursing, Occupational Health, Health Services, Organizational Efficiency

## Abstract

**Objective:**

To characterize presenteeism and productivity losses among nursing professionals in public and private health services.

**Method:**

A cross-sectional study was conducted with 123 participants from a public hospital and 159 from a private hospital in Manaus, Brazil. The Sociodemographic Health Questionnaire was utilized to assess presenteeism, while the Work Limitations Questionnaire measured productivity losses. Data were analyzed using non-parametric methods.

**Results:**

Health-related work loss and presenteeism were reported by 50.41% of public sector professionals and 39.62% of private sector professionals. Despite this, presenteeism was more prevalent in the private sector (93.65%) compared to the public sector. Common health issues included musculoskeletal (26.49%), mental/behavioral (19.21%), respiratory (17.22%), neurological (16.56%), and gastrointestinal (5.96%) conditions. Additionally, 54.24% of private sector professionals and 44.23% of public sector professionals did not seek treatment. The private sector exhibited greater limitations in receiving care due to time management (40.34%), mental-interpersonal tasks (49.95%), production tasks (52.54%), and physical tasks (61.30%), resulting in higher productivity losses among nurses (13.46%) and nursing technicians (15.82%). High-complexity sectors demonstrated the greatest productivity losses.

**Conclusion:**

The study identified significant differences in the characteristics of presenteeism and productivity losses between nursing professionals in the public and private health sectors. These results point to the need to improve management and occupational safety and regulatory measures to solve workers’ health problems and mitigate presenteeism and productivity losses in the public and private health sectors.

## Background

Presenteeism, also known as presenteeism disease, refers to a behavior where individuals with emotional complaints and/or health problems continue to work despite their debilitating conditions. ^(1)^. Although presenteeism is not a recent phenomenon, it remains under-researched and under-recognized by the scientific community and institutions alike. This is problematic because the negative impacts of presenteeism are evident in both worker health and organizational economics across all professional settings.

In terms of overall occupational health, presenteeism significantly deteriorates the physical and mental health of workers, posing a substantial risk factor for future sickness absenteeism and reduced work capacity ^(2,3)^. Economically, presenteeism leads to significant productivity losses, raising costs for organizations, often surpassing those associated with absenteeism and prolonged medical treatment ^(2,4)^. The hidden nature of presenteeism costs stems from extended periods where workers are not performing at full capacity, resulting in long-term losses. Some authors describe presenteeism as an “iceberg effect,” where the visible part of work loss is minor compared to the submerged portion (^5,6)^.

In the global health sector, the World Health Organization (WHO) reports that nursing workers constitute 59% of the health workforce (7). Factors influencing presenteeism in nursing include unfavorable working conditions, high workloads, difficulty in finding replacements, low wages, and neglect of personal health (8, 9). Presenteeism in nursing is linked to compromised quality of care (e.g., work overload, incomplete tasks, demotivation, dissatisfaction) and an increased risk of adverse events (e.g., attention deficits), with medication errors and patient falls being among the most common ^(9, 13,14),^

For instance, the prevalence of presenteeism among nursing professionals in Europe and the United States ranges from 37–83%, resulting in productivity losses of 16.3% and a 12% reduction in workability^1,10)^. Brazil exhibits similar trends in presenteeism prevalence and reduced work capacity among nursing professionals, though productivity losses are higher ^(11,12)^. Despite Brazil’s Unified Health System (SUS, acronym in Portuguese) ensuring universal access, health services are also provided by private entities, either independently or through government contracts. This dual system creates disparities in care, workforce hiring, and work process organization, contributing to varied experiences of presenteeism and productivity losses across public and private sectors.

Efforts to combat presenteeism should focus on improving workplace productivity and promoting health to prevent, minimize, and eliminate health risks, thereby maintaining work capacity ^(15)^. Scientific evidence indicates that workplace health promotion efforts, targeting the physical environment and organizational structure, can positively influence work-related outcomes ^(23)^. Therefore, this study aims to characterize presenteeism and productivity losses among nursing professionals in public and private services in Manaus, Amazonas, to explore specific solutions within the nursing field.

## Methods

This is a cross-sectional observational study conducted from September 2018 to March 2019 in two hospitals in Manaus, AM, Brazil: one federal public hospital and one private hospital. For ethical considerations, the hospital names are omitted.

The federal public hospital, with 159 active beds dedicated to the SUS, provides medium and high-complexity care. Nursing professionals here are hired either under the Unified Legal Regime (ULR), used exclusively by public organizations, or the Consolidation of Labor Laws (CLL), used in both public and private sectors. The private hospital has 121 active beds for patients with direct payment or private health insurance, and it also provides medium- and high-complexity care, with all nursing professionals hired under CLL.

The public hospital employs 207 nursing professionals (94 nurses and 113 nursing technicians), while the private hospital employs 323 (56 nurses and 267 nursing technicians). For equitable comparison, only CLL-hired nursing professionals from the public hospital were considered.

### Statistical Analysis

The stratified probabilistic sampling technique was employed using Epi Info^™^ version 7.2 to calculate the sample size for each participating institution. The calculation considered the following: 1) an expected prevalence of 75%, based on the Work Limitations Questionnaire for Brazilian nursing professionals (16); 2) a 95% confidence level; 3) a 5% margin of error; and 4) a 10% increase to account for losses. To stratify the samples, BioEstat^®^ version 5.3 was used to determine the distribution of strata in each service based on sample and population sizes. The final sample size included 132 participants (60 nurses and 72 nursing technicians) from the public service and 167 participants (29 nurses and 138 nursing technicians) from the private service. Participants were included if they:

1. had worked at least one year in the institution;
2. were not on vacation or leave during data collection;
3. agreed to participate by signing the Free and Informed Consent Form.

The data collection instruments included qualitative interviews with the participants. The sectors and shifts of the study were chosen randomly to avoid bias and ensure an equitable distribution of information. The first instrument, the Sociodemographic and Health Questionnaire (QSS), which was developed for this study and has an English version available, aimed to characterize the study sample by capturing socioeconomic variables related to occupation and health status.

The second instrument, the Work Limitations Questionnaire (WLQ), measures presenteeism by assessing the degree of interference of health problems on work tasks and the resulting impact on productivity ^(17)^. The WLQ was translated and adapted for Brazilian Portuguese ^(18)^. It evaluates physical and/or psychological health issues that occurred in the 14 days prior to completing the self-administered questionnaire, which consists of 25 items grouped into four work limitation domains: 1) Time Management (TM) – measures difficulty in fulfilling schedules and tasks within expected times; 2) Physical Tasks (PhT) – measures ability to perform tasks requiring body strength, endurance, movement, coordination, and flexibility; 3) Mental-Interpersonal Tasks (MIT) – measures difficulty in performing cognitive tasks and interacting with people at work; 4) Production Tasks (PT) – measures difficulty in completing the quantity and quality of work tasks on time ^(18)^.

WLQ scores range from 0 (no limitation) to 100 (limitation all the time), except for the physical tasks domain, which is inversely scored from 0 (limitation all the time) to 100 (no limitation). The WLQ Productivity Loss Index, which indicates the percentage of productivity loss over the past two weeks relative to a healthy sample (maximum productivity loss is 24.9%), is calculated without adjustments for age, sex, or other demographic characteristics ^(18)^.

SPSS Statistics^®^ version 26 was used for statistical analyses, with α = 0.05 and a 95% confidence interval. SDSQ results were presented using absolute and relative frequencies. For the WLQ, the internal consistency of its domains was measured using Cronbach’s alpha coefficient ^(19)^. WLQ data were described using interquartile ranges due to non-normal distribution, verified by the Kolmogorov-Smirnov test.

### Ethical considerations

All participants received written information about the project, had the opportunity to ask the researcher questions and signed a consent form. The questionnaires were completed anonymously. The study was approved by the Research Ethics Committee of the Federal University of the State of Rio de Janeiro, Brazil, under the Certificate of Submission for Ethical Appraisal number: 91296218.7.0000.5285.

### Findings

In the public sector, 95.93% of participants work 36 hours a week, whereas in the private sector, 57.23% work 36 hours, and 40.25% work 40 hours or more. Only 1.63% of public sector professionals and 2.52% of private sector professionals work 30 hours per week. The majority (72.33%) in the private sector have only one employment contract, while a similar percentage (72.35%) in the public sector have more than one bond, resulting in 71.55% of public sector professionals working more than 60 hours per week.

Regarding self-reported monthly income, a large portion (67.92%) of nursing professionals in the private sector earn between USD 260.66 and USD 781.98, whereas 60.17% of public sector professionals earn between USD 1,042.64 and USD 2,345.94. Additionally, only 1.89% of private sector participants reported an income of USD 2,606.60 or more, compared to 24.38% in the public sector.

**Figure 1:**
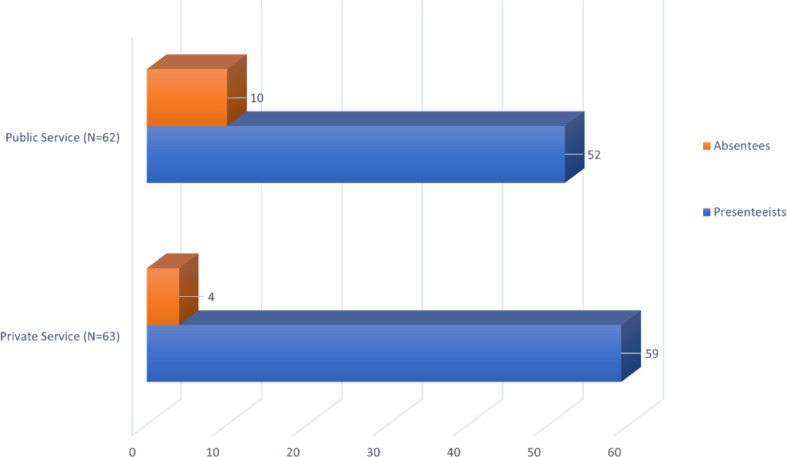
Research participants who reported health problems, according to the prevalence of presenteeism. Manaus/AM, Brazil, 2019.

When asked if they had experienced physical and/or mental health problems in the 14 days prior to the study, 62 (50.41%) participants from the public sector and 63 (39.62%) from the private sector responded affirmatively. Of these, 52 public sector professionals and 59 private sector professionals indicated they had attended work despite being ill. Consequently, the prevalence of presenteeism was 83.87% in the public sector and 93.65% in the private sector (Fig. 1).

The most prevalent health problems reported included musculoskeletal (26.49%), mental/behavioral (19.21%), respiratory (17.22%), neurological (16.56%), and gastrointestinal (5.96%) issues. Despite these problems, a significant portion of participants in both sectors did not seek treatment: 44.23% in the public sector and 54.24% in the private sector. Among those who sought treatment, the majority underwent clinical treatment (67.65% in the public sector and 83.84% in the private sector), followed by mental health treatment (17.65% and 3.33%) and physical therapy (2.94% and 10%).

**Figure 2:**
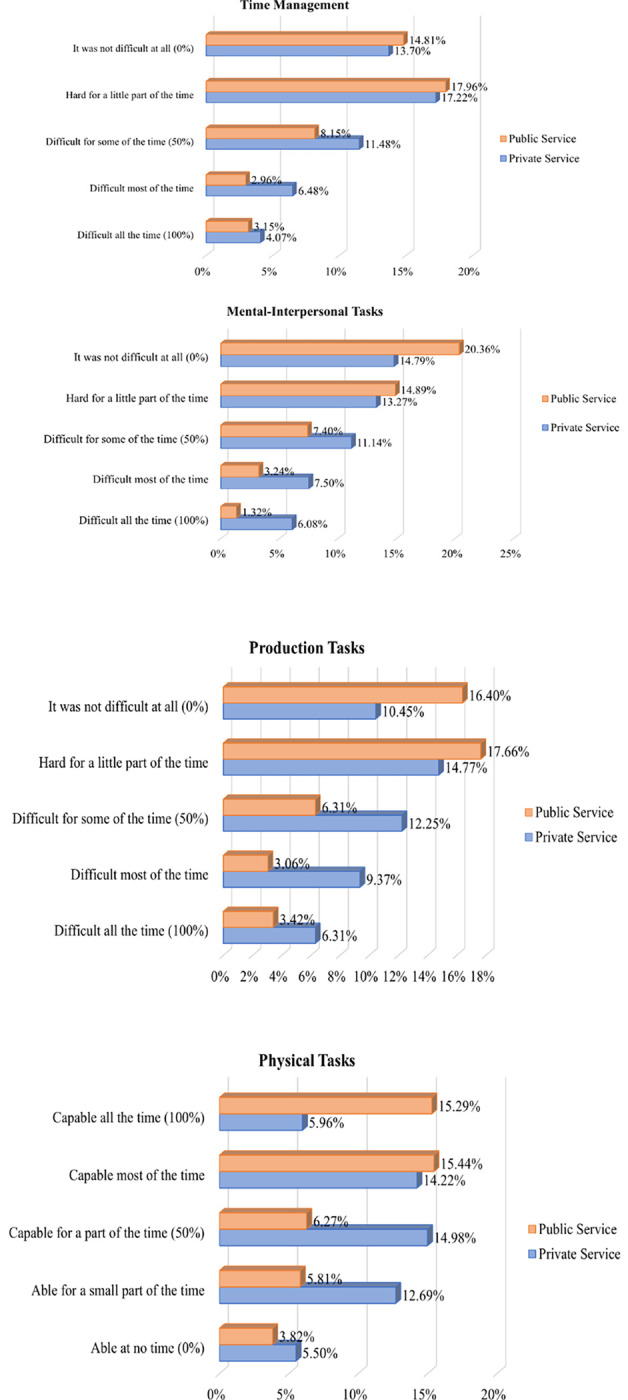
Presenteeism in nursing professionals, according to the frequency of answers* on the domains of the *Work Limitations Questionnaire* - WLQ. Manaus/AM, Brazil, 2019. * Only valid answers were considered

The reliability analysis of the WLQ considered 25 items grouped into four domains and 11 presenteeism variables to verify valid responses. Cronbach’s alpha coefficient ranged from 0.799 to 0.940, indicating good to very good consistency (19). Analysis of participant responses to the WLQ domains (Fig. 2) showed that private sector professionals reported greater work limitations – “part of the time (50%)” to “all the time (100%)” – compared to public sector professionals in Time Management (TM) (40.34% vs. 29.61%), Mental-Interpersonal Tasks (MIR) (62.56% vs. 25.21%), and Production Tasks (PT) (52.54% vs. 27.31%). This trend was also observed in Physical Tasks (PhT), where 61.30% of private sector participants reported greater limitations – “able for part of the time (50%)” to “able at no time (0%)” – compared to 33.33% in the public sector.

As shown in Table 1, in 75% of the private sector sample, the limitation in MIR was up to 56.95% for nurses and up to 62.50% for nursing technicians, while productivity loss was up to 13.46% for nurses and up to 15.82% for nursing technicians. In the public sector, for 75% of the sample, the limitation in PT was up to 45.75% for nurses and in PhT was up to 44.79% for nursing technicians, with productivity losses up to 11.44% for nurses and up to 9.64% for nursing technicians.

In 75% of the private sector sample, the greatest work limitations and productivity losses occurred in the Intensive Care Unit (up to 62.50% in TM, up to 58.33% in PhT, up to 71.88% in MIR, up to 75% in PT, and productivity losses up to 17.16%) and in the Sterile Materials Center (up to 57.92% in PhT, up to 75% in MIR, up to 77.50% in PT, and productivity losses up to 17.35%). In the public sector, the greatest work limitations and productivity losses occurred in the Surgical Center (up to 72.50% in TM, up to 52.78% in MIR, up to 72.50% in PT, and productivity losses up to 15.92%) and in the Sterile Materials Center (up to 80% in TM, up to 74.31% in MIT, up to 92.50% in PT, and productivity losses up to 19.43%).

## Discussion

The prevalence of presenteeism among nursing professionals described in this study exceeded those previously observed in both national(^9,11)^ and international^(1,8)^ studies. This demonstrates that presenteeism, despite being an old issue, is continuously growing.

In the public sector, many participants had more than one job, which meant that they exceeded the 60-hour working week limit set by the Superior Court of Justice. This limit is important to “safeguard the physical and mental integrity of the worker and the efficiency of the work” (11, 13). Multiple employment contracts and excessive weekly working hours contributed to the higher percentage of illness among public service workers.

However, presenteeism was more prevalent in the private sector. This could be attributed to the fact that, unlike private sector workers, public sector workers in Brazil enjoy a higher degree of job protection (e.g., job stability, better wages, prolonged leave without salary losses), which influences their decision between missing work due to illness (absenteeism) or attending work while sick (presenteeism). One study^(12)^ indicates that the likelihood of presenteeism among nursing professionals with permanent employment was 112% higher. Nevertheless, the patterns of absenteeism and presenteeism vary according to the health condition of the workers, influenced by various organizational and individual factors.

In this study, the prevalence of presenteeism was higher among nursing professionals in the private sector, who reported lower incomes compared to their public sector counterparts. This finding aligns with other studies ^(22),^ which observed that most presenteeism workers had low income, low education, and seasonal work contracts, suggesting a relationship between monthly income and presenteeism.

Organizational culture also plays a role in presenteeism, particularly regarding the emphasis on workplace presence. Many policies aimed at reducing absenteeism (e.g., awards, benefits, promotions) can inadvertently encourage presenteeism. In such cases, workers may stay on the job as long as possible, potentially suffering greater health damage ^(20)^. Additionally, workers interested in salary increases, bonuses, promotions, or maintaining their reputation may engage in “competitive presenteeism” as a way to “make a presence” in the organization ^(21)^.

The health problems reported in this study are consistent with those found in other national ^(9,12)^ and international ^(10,13)^ studies, indicating a trend of health issues related to presenteeism among nursing professionals..

Regarding the treatment of reported health problems, most participants in both public and private sectors did not seek treatment. This is concerning, as remaining untreated can lead to further health deterioration, reduced work capacity, increased absenteeism, overburdening colleagues, and compromising the quality of nursing care and patient safety ^(5,13)^. The higher percentage of untreated health issues in the public sector may reflect a lack of monitoring or insufficient monitoring of workers’ health and patient care.

For those who sought treatment, the majority underwent clinical treatment, with fewer seeking mental health or physiotherapeutic treatment, despite the high prevalence of musculoskeletal and mental/behavioral disorders. This highlights the need for early diagnosis of workers’ health problems to better direct occupational health actions.

Due to the high prevalence of musculoskeletal and mental/behavioral disorders, the non-adherence to treatment, and the low demand for mental and physiotherapeutic treatment, private sector participants reported greater work limitations in all domains of the WLQ and, consequently, greater productivity losses among both nurses and nursing technicians compared to the public sector.

In the private sector, the Intensive Care Unit and the Sterile Materials Center exhibited the greatest work limitations and productivity losses, while in the public sector, this was observed in the Surgical Center and the Sterile Materials Center. The high technological density and repetitive tasks in these sectors likely contribute to the increased workload, stress, and strain on nursing professionals compared to units handling low/medium complexity or technological density health services. These findings differ from other national studies ^(17,21),^ which found greater work limitations and productivity losses in open sectors that do not require intensive specialization. This indicates that Brazilian nursing workers are experiencing worsening health conditions and that presenteeism is on the rise.

## Conclusion

In Brazil, the nursing field is characterized by multiple work bonds, low wages, long working hours, late retirement, and precarious working conditions. Despite these challenges, nursing professionals are more likely to work while sick (presenteeism) than to miss work (absenteeism). Therefore, it is crucial to study the characteristics of presenteeism in Brazilian nursing, investigate related factors, and quantify productivity losses at work.

This study highlighted that nursing professionals’ behaviors in response to illness vary depending on their work relationship. Public sector professionals are more prone to absenteeism, while private sector professionals exhibit higher rates of presenteeism. Factors contributing to this include the protectionism of the public service, working conditions, job flexibility, and precariousness, as well as an organizational culture that combats absenteeism but inadvertently encourages presenteeism.

As a result, the greatest work limitations and productivity losses were observed among nursing workers in the private sector. Professionals working in high-complexity, closed sectors presented the highest rates of work limitations and productivity losses. These findings necessitate urgent attention from managers for diagnosis, planning, and implementation of actions to combat presenteeism through occupational health programs.

This study aims to provide data that can guide preventive, promotive, and restorative health actions for these workers through Occupational Health and Safety Programs. These programs could include initiatives such as ergonomics, mental health support, vaccinations, use of personal protective equipment, creation of a healthy work environment, occupational gymnastics, noise reduction, and adequate nutrition, among others.

### Limitations

This study was limited to nursing professionals governed by the CLL. Therefore, additional studies are necessary to compare the characteristics of presenteeism between statutory servants (from federal, state/district, and municipal levels) and other public employees.

## Figures and Tables

**Table 1 -:** Score of the WLQ* and WLQ* Productivity Loss Index domains, according to the professional category of the participants. Manaus, AM, Brazil, 2019.

Professional Categories / Domains and WLQ Index*	Public Service (%)			Private Service (%)		
	25†	50‡	75§	25†	50‡	75§
**Nurses**						
Time Management	15	25	50	13.75	32.50	52.50
Physical Tasks	11.25	25	40.63	22.92	31.25	50
Mental-Interpersonal Tasks	11.11	18.06	41.66	15.28	25	56.95
Production Tasks	10	20	46.75	32.50	37.50	48.25
WLQ Productivity Loss Index	3.96	5.92	11.44	6.93	8.63	13.46
**Nursing Technicians**						
Time Management	7.19	20	33.75	17.50	30	47.50
Physical Tasks	12.50	31.25	44.79	27.09	45.83	56,67
Mental-Interpersonal Tasks	5.56	13.89	24.31	12.50	32.14	62.50
Production Tasks	10	20	43.75	20	45	70
WLQ Productivity Loss Index	2.86	5.06	9.64	5.42	10.90	15.82

* WLQ = *Work Limitations Questionnaire*

† 1st quartile,

‡ median and

§ 3rd quartile

**Table 2 -:** Score of the WLQ* and WLQ* Productivity Loss Index domains, according to the work sector of the participants. Manaus, AM, Brazil, 2019.

WLQ Domains and Index* / Labor Sectors	Civil service			Private Service		
	25†	50‡	75§	25†	50‡	75§
**Time Management**						
Outpatient	3.75	20	28.75	11.25	22.50	22.50
Administrative/Advisory	0	7.50	11.25	18.75	30	26.25
Inpatient Units	13.75	20	32.50	14.38	27.50	40
Surgical Center	21.25	30	72.50	14.38	17.50	52.50
Sterile Materials Center	8.13	40	80	20	35	40
Intensive Care Unit	20.31	30	50	15	40	62.50
**Physical Tasks**						
Outpatient	2.08	10.42	25.63	18.75	33.34	31.25
Administrative/Advisory	6.5	31.25	40.63	15.62	39.58	43.75
Inpatient Units	21.25	39.59	57.29	21.87	43.75	57.29
Surgical Center	4.17	29.17	39.59	19.79	35.42	50
Sterile Materials Center	13.75	20.83	41.67	30	37.50	57.92
Intensive Care Unit	21.87	33.34	48.96	25	45.83	58.33
**Mental-Interpersonal Tasks**						
Outpatient	3.47	8.33	15.28	6.25	31.95	41.67
Administrative/Advisory	2.09	2.78	2.09	8.33	13.89	12.50
Inpatient Units	9.72	16.67	30.56	8.33	26.39	48.61
Surgical Center	11.81	15.28	52.78	7.64	19.45	45.75
Sterile Materials Center	9.72	44.44	74.31	25	56.25	75
Intensive Care Unit	11.81	18.06	41.66	16.67	37.50	71.88
**Production Tasks**						
Outpatient	2.0	12.50	18.75	18.75	40	41.25
Administrative/Advisory	7.50	10	7.50	7.50	17.50	18.75
Inpatient Units	10	20	40	6.25	27.50	53.75
Surgical Center	10	17.50	72.50	10	35	51.25
Sterile Materials Center	15	40	92.50	37.50	45	77.50
Intensive Care Unit	2.50	22.50	48.75	25	50	75
**WLQ Productivity Loss Index**						
Outpatient	1.57	3.19	5.11	3.71	9.05	9.86
Administrative/Advisory	1.21	2.77	2.94	2.99	5.86	5.79
Inpatient Units	3.45	5.37	9.44	3.43	7.91	11.91
Surgical Center	3.94	5.78	15.92	4.41	7.52	13.4
Sterile Materials Center	3.44	11.11	19.43	8.45	12.24	17.35
Intensive Care Unit	3.45	6.72	11.74	6.77	13.39	17.16

* WLQ = *Work Limitations Questionnaire*

† 1st quartile,

‡ median and

§ 3rd quartile

## Data Availability

Data will be available on reasonable request from the corresponding author.

## Abbreviations

CLL: Consolidation of Labor Laws
MIT: Mental-Interpersonal Tasks
PhT: Physical Tasks
SDSQ: Sociodemographic and Health Questionnaire
SPSS: Statistical Package for the Social Sciences
SUS: Unified Health System
TM: Time Management
ULR: Unified Legal Regime
WHO: World Health Organization
WLQ: Work Limitations Questionnaire
